# Prevalence and influence factor of drug-related problems in inpatients with kidney disease: a prospective single central study

**DOI:** 10.3389/fphar.2024.1436561

**Published:** 2024-10-30

**Authors:** Guoguang Gu, Yanping Li, Yunyun Hu, Hengyi Zhao, Xingdong Wang, Xiaomin Li, Xinran Zhang, Hong Zhu, Xiaohua Dai, Xingxing Liu, Liyan Miao, Jianguo Zhu, Yongfu Hang

**Affiliations:** ^1^ Department of Pharmacy, The First Affiliated Hospital of Soochow University, Suzhou, China; ^2^ Department of Pharmacy, The First affiliated Hospital of Xiamen University, School of Medicine, Xiamen University, Xiamen, China; ^3^ Department of Pharmacy, Suzhou Xiangcheng People’s Hospital, Suzhou, China; ^4^ Department of Pharmacy, Xuzhou Central Hospital, Xuzhou, China; ^5^ Department of Pharmacy, The First Hospital of Lanzhou University, Lanzhou, China; ^6^ Department of Pharmacy, ZhongDa Hospital, School of Medicine, Southeast University, Nanjing, China; ^7^ Department of Pharmacy, Tianjin First Central Hospital, Tianjin, China; ^8^ Department of Pharmacy, Suzhou Kowloon Hospital Affiliated to School of Medicine, Shanghai Jiaotong University, Suzhou, China; ^9^ Department of Pharmacy, Zhaoqing First People’s Hospital, Zhaoqing, China; ^10^ Department of Pharmacy, Guiyang Maternal and Child Healthcare Hospital, Guiyang, China

**Keywords:** kidney disease, drug-related problems (DRPs), medication reconciliation, clinical pharmacist, renal anemia

## Abstract

**Aims:**

To investigate the prevalence and influencing factors of drug-related problems (DRPs) in inpatients with kidney disease to provide reference data for pharmaceutical care.

**Methods:**

The basic information, diagnoses, and medication reconciliation (MR) of inpatients in the Department of Nephrology at our hospital between October 2020 and September 2021 were collected. The Chinese-modified DRP version based on the PCNE classification (Version 9.1) was used to assess, intervene and statistically analyze the results of the patients’ DRPs. The influence factor of DRPs in inpatients with kidney disease was analyzed by the multivariate binary logistic regression.

**Results:**

Of 623 patients included in this study, 132 (21.80%) had DRPs. The prevalence of anemia was significantly higher in patients with DRPs than those without DRPs (43.18% vs. 28.72%, *p* < 0.05), the mean number of drug types consumed (7.25 ± 3.44 with DRPs vs. 5.93 ± 3.58 without DRPs, *p* < 0.05) and the proportion of ≥5 drugs (%) (79.55% with DRPs vs 58.04% without DRPs, *p* < 0.05) were significantly increased. In addition, the prevalence of hypertension (76.52% vs. 68.64%), diabetes (27.27% vs. 22.20%) and hyperuricemia (16.67% vs. 13.65%) in DRP patients were higher than those without DRPs, but there was no statistical difference (*p* > 0.05). The logistic regression analysis showed that patients with anemia (OR = 1.702, 95%CI: 1.146–2.529, *p* = 0.008), average number of medication types taken (OR = 1.089, 95%CI: 1.034–1.147, *p* = 0.001) significantly increased the risk of DRPs. The distribution of harm levels was as follows: 78 problems (59.09%) were level C, 29 (21.97%) were level B, 10 (7.58%) were level D, 7 (5.30%) were level A, 7 (5.30%) were level E, and 1 (0.76%) were level F. All DRPs were resolved after 128 interventions.

**Conclusion:**

Renal anemia, the average number of drug varieties consumed, and the proportion of ≥5 drugs are associated with the occurrence of DRPs. Pharmacists conducting MR services can reduce DRPs of inpatients in the department of nephrology and ensure patient drug safety.

## Introduction

Nephrology is a relatively complex and special department concerning medication use. Renal disease is frequently accompanied by hypertension and diabetes mellitus, among other chronic conditions; therefore, the patient’s conditions are more complicated, requiring the use of multiple medications and increasing the risk of drug interactions. The dose of medicine should be adjusted according to the patient’s renal function and administered with great care (Hatano et al., 2022). The increase in the quantity and diversity of medications consumed by patients not only decreases compliance but also elevates the prevalence of drug-related problems (DRPs). The results of the systematic review show that DRPs are more common in patients with stage 1–4 chronic kidney disease (CKD), and have brought a large economic burden (Alruqayb et al., 2021). These studies have shown that factors associated with DRPs include the severity of CKD, length of hospital stay, number of medications taken, age, and gender (Alruqayb et al., 2021), but there is a lack of supporting data from the Chinese population.

Medication reconciliation (MR) is a standardized pharmacy service in which the present medication regimen of a patient is cross-referenced with the medication order to resolve medical deviations or potential medication problems. MR conducted by a clinical pharmacist has a reduced error rate compared to that of physicians. This not only conserves healthcare resources but also ensures the accuracy and continuous administration of medication therapy (Joint Commission on Accreditation of Healthcare OrganizationsUSA, 2006). Pharmacist-led MR in nephrology is well-established overseas and has been previously reported in China (Wang et al., 2018; Yu et al., 2020). China’s MR has been implemented late and lacks a standardized and mandatory working mechanism with an apparent division of labor. Patients with kidney disease are at an increased risk for DRPs, therefore, MR in this population is of critical importance (Joint Commission on Accreditation of Healthcare OrganizationsUSA, 2006). Consequently, in the present study, we employed the previously investigated MR model (Wang et al., 2018; Yu et al., 2020; Zhao et al., 2020) to analyze and intervene in the medication use of nephrology inpatients at our hospital. Our objective was to provide clinical pharmacists with a base and reference for further adjusting their services and to better understand the prevalence of DRPs among nephrology inpatients.

## Materials and methods

### Sources of information

The data were obtained from patients admitted to the nephrology department of our hospital between October 2020 and September 2021. The inclusion criteria were as follows: (i) use of ≥1 drug and (ii) ability to communicate normally. The exclusion criteria were as follows: (i) patients who were unwilling to cooperate with the clinical pharmacist and (ii) those with incomplete information.

### Research methodology

Within 48 h of the patient’s admission to the nephrology unit, the clinical pharmacist obtained a complete and accurate medication history, including the name, specifications, duration of treatment, dosage, and any severe adverse reactions, by conducting an MR with the patient and their family. The clinical pharmacist evaluated the patient’s medication compliance, determined the existence of DRPs, and updated the Chronic Kidney Disease Management Manual medication list, which includes the names of all medications currently taken by the patient, the purpose of the drug, dosage, precautions, start and stop times of the medicine, and others.

### Assessment criteria

#### Adherence assessment

The Morisky Medication Adherence Scale (MMAS-8) evaluated the patient’s adherence to their medication; it contains 8 questions; the details can be found in the Supplementary Table S1. A score of 8 indicated good patient adherence, 6–7 indicated moderate patient adherence, and <6 indicated poor patient adherence (Yan, et al., 2014).

#### Chinese version of the DRP classification system

In the context of the Chinese healthcare environment and practice, we adapted the classification of DRPs (version 9.1) proposed by the Pharmacovigilance Consortium of Europe (PCNE) (Pharmaceutical Care Network Europe, 2020). Presently, pharmacists in China do not have prescribing authority and cannot directly change or adjust patients’ medication regimens. Therefore, we have changed the term “drug level” in the intervention (I) to “drug level intervention with the consent of the physician.” The original DRPs lacked potential harm assessment; therefore, we innovatively combined the potential harm assessment of medication errors (4 levels and 9 classifications) (Cousins and Heath, 2008) in the Chinese Expert Consensus on Medication Error Management (International Network for Rational Drug Use China Central Group clinical Safe Drug Use GroupChinese Pharmacological Society Of Drug-induced Diseases Professional CommitteeChinese Pharmaceutical Society of Hospital Pharmacy professional Committeeetc, 2014) with the classification of DRPs, forming six aspects: problem (P–), cause (C–), intervention (I–), acceptance (A–), status (O–), and potential harm assessment (ME). Thus, DRPs were identified, analyzed, intervened, followed up, and documented, forming a closed loop for each DRP, resulting in a modified version of the DRP classification system.

#### Statistical analysis

In this study, the data were statistically analyzed using Statistical Package for the Social Sciences (SPSS) (version 25). The measurement data were expressed as x̄ ± s using a *t*-test; the count data were expressed as composition ratio (%) using χ^2^ test. The risk of presenting DRPs was modeled with the results obtained using a multivariate binary logistic regression model. *p* < 0.05 was considered a statistically significant difference.

## Results

### General information about the patient

**FIGURE 1:**
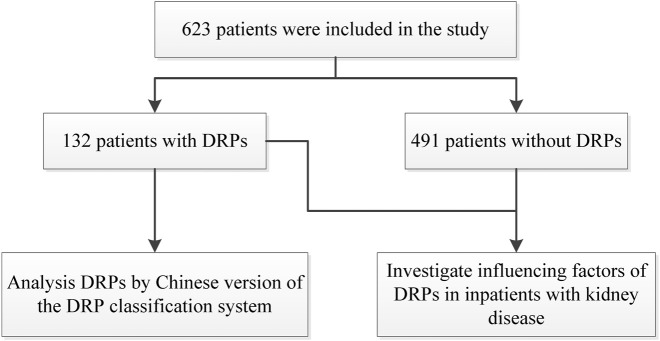
The flow diagram of study.

623 patients were finally included in the study (Figure 1), including 328 males and 295 females, with a mean age of 52.27 ± 16.82 years (18–87 years) and 182 cases (29.21%) aged ≥65 years. Among them, based on the CKD stage, 10 cases (1.61%) were in stage 2, 72 (11.56%) in stage 3, 51 (8.19%) in stage 4, and 206 (33.07%) in stage 5. Among the patient’s underlying diseases, hypertension was the most common, with 438 cases (70.30%), followed by anemia in 198 (31.78%), diabetes mellitus in 145 (23.27%), hyperuricemia in 89 (14.29%).

### Prevalence of DRPs

#### General profile of patients with DRPs

DRPs were present in 132 of the 623 cases, with an prevalence of 21.8% (132/623). Out of 132, 55 were female and 77 were male. Although the percentage of patients aged 65 and older and the mean age of patients with DRPs were both higher than those without DRPs, no statistically significant difference was observed (*p* > 0.05). Out of 623 cases, 339 patients (54.41%) had CKD, and 80 patients with CKD had DRPs, representing an prevalence of 23.60%. Sixteen patients (12.12%) were classified as having CKD stage 3, fourteen (10.61%) having CKD stage 4, and 50 (37.88%) having CKD stage 5. Patients with DRPs had a substantially higher prevalence of anemia than those without DRPs (43.18% vs. 28.72%, *p* < 0.05). The logistic regression analysis (detailed logistical regression shown in Supplementary Table S2) showed that the significant correlation between DRPs and the prevalence of anemia (OR = 1.702, 95% CI: 1.146–2.529, *p* = 0.008), average number of medication types taken (OR = 1.089, 95% CI: 1.034–1.147, *p* = 0.001). Additionally, the prevalence of hypertension (76.52% vs. 68.64%), diabetes mellitus (27.27% vs. 22.20%), and hyperuricemia (16.67% vs. 13.65%) was higher in patients with DRPs than those without DRPs. However, none of them were statistically different (*p* > 0.05) (Table 1).

**TABLE 1:** Comparison of people with and without DRPs.

Projects	DRPs occur	No DRPs occurred	*p*-value
Number of people	132	491	—
Average age	53 ± 16.59	52.11 ± 16.92	0.587
Number of people aged ≥65 years (%)	44 (33.33%)	138 (28.11%)	0.241
High blood pressure	101 (76.52%)	68.64%	0.079
Anemia	57 (43.18%)	28.72%*	0.002
Diabetes	36 (27.27%)	22.20%	0.221
Hyperuricemia	22 (16.67%)	13.65%	0.379
Average number of medication types taken	7.25 ± 3.44	5.93 ± 3.58*	<0.001
Percentage of people with ≥5 drugs (%)	105 (79.55%)	285 (58.04%)*	<0.001

*P < 0.05.

Of the 132 patients with DRPs, the mean number of drugs taken per patient was (7.25 ± 3.44), significantly more than those without DRPs (5.93 ± 3.58) (*p* < 0.05); a significantly higher proportion of patients with DRPs took ≥5 drugs (*p* < 0.05) (Table 1). A total of 132 cases were mainly involved in drug classes, and frequencies are shown in Table 2.

**TABLE 2:** Summary of medication reorganization list for patients with DRPs.

Drug class	Frequency (%)
Antihypertensive drugs	259 (27.04)
Kidney Disease Support	162 (16.91)
Anti-anemic drugs	120 (12.53)
Calcium and phosphorus-regulating drugs	72 (7.52)
Nutritional medicine	68 (7.10)
Glucose-lowering drugs	68 (7.10)
Uric acid-lowering drugs	55 (5.74)
Lipid-regulating drugs	37 (3.86)
Antithrombotic drugs	34 (3.55)
Other	29 (3.03)
Acid-suppressing and gastrointestinal stimulants	26 (2.71)
Psychotropic depressants	16 (1.67)
Liver-protective drugs	12 (1.25)
Total	958 (100)

#### Categories of patients with DRPs and reasons for them

As shown in Table 3, the majority of patients with DRPs (n = 80, or 60.61%) had DRPs associated with treatment safety, while 35 (26.52%) had DRPs related to treatment efficacy, and 17 (12.88%) had DRPs for other conditions (Table 3). 132 DRPs can be classified into 133 problem categories: 77 in terms of the use of medication, 51 for prescribing, and five in terms of dispensing. All of the other causes in C9.2 were more specific cases where the patient’s medication was reasonable, however, the actual medical advice was wrong or not prescribed, as shown in Table 4.

**TABLE 3:** Categories of DRPs.

Categories of DRPs	Number of cases/case	Proportion/%
P1 Treatment effectiveness	35	26.52
P1.1 No effect of drug treatment	1	0.76
P1.2 Effect of drug treatment not optimal	29	21.97
P1.3 Untreated symptoms or indication	5	3.79
P2 Treatment safety	80	60.61
P2.1 Adverse drug event (possibly) occurring	80	60.61
P3 Others	17	12.88
P3.1 Problem with the cost-effectiveness of the treatment	5	3.79
P3.2 Unnecessary drug-treatment	8	6.06
P3.3 Unclear problem/complaint. Further clarification necessary (please use as an escape only)	4	3.03
Total	132	100.00

**TABLE 4:** Causes of DRPs.

Categories of causes	Number of cases/case	Proportion/%
Prescribing and drug selection	51	38.35
C1 Drug selection	26	19.55
C1.1 Inappropriate drug according to guidelines/formulary	14	10.53
C1.2 No indication for drug	2	1.50
C1.3 Inappropriate combination of drugs, or drugs and herbal medications, or drugs and dietary supplements	2	1.50
C1.4 Inappropriate duplication of the therapeutic group or active ingredient	5	3.76
C1.5 No or incomplete drug treatment despite existing indication	3	2.26
C3 Dose selection	25	18.80
C3.1 Drug dose too low	4	3.01
C3.2 Drug dose of a single active ingredient too high	9	6.77
C3.3 Dosage regimen not frequent enough	3	2.26
C3.4 Dosage regimen too frequent	8	6.02
C3.5 Dose timing instructions wrong, unclear, or missing	1	0.75
Disp	5	3.76
C5 Dispensing	5	3.76
C5.2 Necessary information not provided or incorrect advice provided	5	3.76
Use	77	57.89
C7 Patient related	42	31.58
C7.1 Patient intentionally uses/takes less drug than prescribed or does not take the drug at all for whatever reason	18	13.53
C7.3 Patient abuses drug (unregulated overuse)	1	0.75
C7.4 Patient decides to use an unnecessary drug	2	1.50
C7.7 Inappropriate timing or dosing intervals	17	12.78
C7.8 Patient unintentionally administers/uses the drug in a wrong way	4	3.01
C8 Patient transfer-related	11	8.27
C8.1 Medication reconciliation problem	11	8.27
C9 Other	24	18.05
C9.1 No or inappropriate outcome monitoring (incl. TDM)	1	0.75
C9.2 Other cause; specify	20	15.04
C9.3 No obvious cause	3	2.26
Total	133	100.00

#### Types of interventional programs and acceptance of DRP

The clinical pharmacist executed 128 interventions for DRPs that occurred in the nephrology department’s inpatients (Table 5). Of these, 78 (17.09%) were attributable to the physician level, 23 (17.42%) to the patient level, 24 (18.18%) to the medication level, and 3 (2.27%) to other interventions or behaviors. Patients were able to accept the medication reorganization protocols that the clinical pharmacist delivered through medication guidance and interventions. The acceptance rate for the intervention protocols and implementation was 100%, and all issues were resolved.

**TABLE 5:** Types of intervention programs for DRPs.

Types of intervention programs	Number of cases/case	Proportion/%
I0.1 No Intervention	4	3.03
I1 At the prescriber level	78	59.09
I1.1 Prescriber informed only	19	14.39
I1.2 Prescriber asked for information	1	0.76
I1.3 Intervention proposed to prescriber	52	39.39
I1.4 Intervention discussed with prescriber	6	4.55
I2 At the patient level	23	17.42
I2.1 Patient (drug) counseling	7	5.30
I2.4 Spoken to family member/caregiver	16	12.12
I3 At drug level with the consent of the physician	24	18.18
I3.2 Dosage changed to …	3	2.27
I3.4 Instructions for use changed to …	17	12.88
I3.5 Drug paused or stopped	4	3.03
I4 Other intervention or activity	3	2.27
I4.2 Side effect reported to authorities	3	2.27
Total	132	100.00

#### DRP potential hazard assessment

Potential hazards were assessed for 78 (59.09%) of the 132 DRPs that occurred; Class C comprised the largest number, followed by Class B with 29 (21.97%), Class D with 10 (7.58%), Class A and E with 7 (5.30%), and Class F with 1 (0.76%).

## Discussion

The nephrology department exhibited a 21.8% prevalence of DRPs. Compared to patients without DRPs, those with DRPs had substantially higher rates of combined anemia, the mean number of medication types, and ≥5 medications (%). This suggested that the prevalence of DRPs was strongly associated with the number of medication types taken. The prevalence of renal anaemia rises progressively as renal function declines further. Patients with renal anaemia are often on iron supplements due to absolute iron deficiency, along with roxadustat. Calcium and the phosphorus-lowering drug sevelamer are also used concurrently. The majority of patients concurrently administer these medications, which necessitates periodic dosing, which could create many potentially adverse drug-drug interactions and DRPs. This can also lead to poor correction of anaemia. Although not statistically significant, patients with DRPs had a higher mean age, a higher proportion of combined underlying diseases of all types, and a higher mean number of drug varieties per patient than those without DRPs. A total of 339 patients had CKD (54.41%) out of 623 cases, and a total of 80 patients with CKD had DRPs, with an prevalence of 23.60%. Another study of DRPs in our hospital in 2017–2018 in patients with inpatient CKD enrolled 113 patients, and the prevalence of DRPs was 77% (Liu et al., 2021). The significant decrease in the occurrence of DRPs among patients with CKD could be attributed to the extended duration of the study and the broader range of services included in this research. Additionally, implementing medication reorganization in our hospital could potentially be linked to this.

Treatment safety accounted for most patients with DRPs, comprising 80 entries (60.61%), all of which fell under the category of (possible) adverse drug events. ADRs occurred in 60 of these patients during use, or they encountered issues with repeated medication, contraindications to dosing, or unreasonable dosage. These complications may be attributed to the following factors: the average age of patients with DRPs, the high proportion of patients aged ≥65 years, stage 5 CKD, more comorbid underlying conditions, and poorer adherence. A total of 35 patients (26.52%) with DRPs correlated with treatment efficacy. The absence of professional oversight regarding medication use before hospitalization substantially elevates the risk of medication use, particularly among patients with CKD who lack adequate knowledge of disease progression, fail to adequately monitor it, frequently administer medication randomly, and miss medication.

Regarding causes, 57.89% of DRPs were attributable to the process of use of medications, of which 31.58% were related to patient use. In 13.53% of cases, “the patient deliberately reduced the dose or did not take the medication at all for various reasons,” i.e., Some patients will intentionally reduce the dosage of Compound α-ketoacid tablets for financial reasons. Furthermore, some patients reduce the dosage of antihypertensive drugs without doctor’s advice, concerned that an excessively high dosage may lead to drug resistance and a subsequent depletion of therapeutic options. In addition, in 12.78% of cases, “the patient took the medication at an inappropriate time or interval.” i.e., Many patients take iron and calcium, polyvalent cations and roxarrestat and sevelamer together, ignoring the fact that these drugs need to be taken over a long period. This highlights the fact that the risk of patients taking medication due to improper medication cannot be ignored. Moreover, 38.35% of cases were related to prescriptions, of which drug selection and dose selection accounted for nearly 20%, indicating that the rationality of drug selection and dose adjustment in prescriptions for patients with renal disease should not be ignored and that prior review of prescriptions needs to be further strengthened.

In our study, “other reasons” comprised 15.04% of the cases, i.e., 20 patients were found to be taking the correct medication according to their condition. Nevertheless, the existing medical advice was inaccurate or non-prescribed, demonstrating the complexity of the deviation between clinical advice and the patient’s actual usage. This may result in the clinician incorrectly assessing the effectiveness of the current treatment plan. Such deviations are likely to cause the clinician to misjudge the efficacy of the current treatment regimen, thereby compromising treatment. This incident has also been detected for the first time, emphasizing the need for medication reorganization.

In the study of the types of interventions for DRPs, 17.42% of the interventions were at the “patient-level,” and most patients were able to accept the recommendations, and the DRPs were resolved. This demonstrated that medication instruction and education are essential steps in improving adherence to medical advice and illustrates the importance of medication education in ensuring patient adherence to medication. The percentage of interventions at the “physician level” was 59.09%, and the percentage of interventions provided to physicians was 39.99%, with 100% of interventions accepted and fully implemented, which also shows that clinical pharmacists can effectively prevent clinical medication errors through continuous interventions on unreasonable medical advice. In the hazard assessment of DRPs, 86.36% did not cause harm to patients; however, 7.58% of grade D needed to be closely monitored, and 6.06% of grades E and F had caused some harm to patients, which physicians and pharmacists must pay high attention.

The high prevalence of DRPs in kidney disease patients is a significant challenge as it influences the morbidity, mortality, and quality of life of these patients.

In this work, we investigated the prevalence and influencing factors of drug-related problems (DRPs) in inpatients with kidney disease, and found Renal anemia, the average number of drug varieties consumed, and the proportion of ≥5 drugs are associated with the occurrence of DRPs. This will help to screen priority populations and improve the efficiency of pharmacy services in the future. There were some limitations in our study. For example, it was conducted in single center. While we have considered factors such as age, we did not included other variables such as dietary lifestyle, level of education or literacy, knowledge about the drugs, profession, and the duration of hospitalization. Given that the average length of stay was generally less than 10 days, no statistical analysis regarding the duration of hospitalization was conducted. Patient adherence was assessed solely at the time of admission. Furthermore, no subsequent assessments of adherence were performed. This represents a limitation of the current study, which will be addressed in future research endeavors.

## Conclusion

Our results confirmed the findings of our earlier research that DRPs in kidney disease inpatients are common. Renal anemia, the average number of drug varieties consumed, and the proportion of ≥5 drugs are related to the occurrence of DRPs. Medication reconciliation decreased the number of DRPs and promoted medication safety. Clinical pharmacist interventions were highly accepted by physicians and patients. This may indicate that the implementation of MR services in the nephrology ward was essential and also had a significant impact on optimized therapy and prevention DRPs.

## Data Availability

The datasets from the current study are not publicly available due to their containing information that could compromise the privacy of research participants, but are available from the corresponding author on reasonable request. Requests to access these datasets should be directed to hangyongfu1986@163.com.

## References

[B1] Alruqayb W. S. Price M. J. Paudyal V. Cox A. R. (2021). Drug-related problems in hospitalised patients with chronic kidney disease: a systematic review. Drug Saf. 44 (10), 1041–1058. 10.1007/s40264-021-01099-3 34510389

[B2] Cousins D. D. Heath W. M. (2008). The national coordinating council for medication error reporting and prevention: promoting patient safety and quality through innovation and leadership. Jt. Comm. J. Qual. patient Saf. 34 (12), 700–702. 10.1016/s1553-7250(08)34091-4 19119722

[B3] Hatano M. Mizuno T. Arakawa Y. Inagaki R. Kato A. Matsuzaki H. (2022). Efficacy of a pharmacist team clinical medication review in older adults: a prospective and retrospective observational study. Biol. and Pharm. Bull. 45 (8), 1166–1171. 10.1248/bpb.b22-00245 35908897

[B4] International Network for Rational Drug Use China Central Group clinical Safe Drug Use Group, Chinese Pharmacological Society Of Drug-induced Diseases Professional Committee, Chinese Pharmaceutical Society of Hospital Pharmacy professional Committee, etc (2014). Expert consensus on medication error management in China. J. adverse drug React. 16 (6), 321–326. 10.3760/cma.j.issn.1008-5734,2014.06.001

[B5] Joint Commission on Accreditation of Healthcare Organizations, USA (2006). Using medication reconciliation to prevent errors. Sentin. event alert 35, 1–4. 10.1007/s40264-021-01099-3 16933570

[B6] Liu X. X. Wang H. X. Hu Y. Y. Zhu X. T. Tan X. Yang Y. (2021). Drug-related problems identified by clinical pharmacists in nephrology department of a tertiary hospital in China-a single center study. Ann. Palliat. Med. 10 (8), 8701–8708. 10.21037/apm-21-817 34488359

[B7] Pharmaceutical Care Network Europe (PCNE) (2020). The PCNE classification V9.1. Available at: https://www.pcne.org/upload/files/449_2021-01-27_Report_online_validation_PCNE_classification_v9.1.pdf (Accessed October 5, 2020).

[B8] Wang H. X. Zhu X. T. Xie C. Qian Y. L. Sun S. S. Hang Y. F. (2018). Preliminary study on drug restructuring service for patients with chronic kidney disease by clinical pharmacists in our hospital. Chin. J. Pharm. 53 (24), 2148–2151. 10.11669/cpj.2018.24.017

[B9] Yan J. You L. M. Yang Q. H. Liu B. L. Jin S. Y. Zhou J. J. (2014). Translation and validation of a Chinese version of the 8-item Morisky medication adherence scale in myocardial infarction patients. J. Eval. Clin. Pract. 20 (4), 311–317. 10.1111/jep.12125 24813538

[B10] Yu W. L. Liu X. X. Lu R. L. Liu X. Shen L. Hang Y. F. (2020). Practice of clinical pharmacists participating in joint outpatient service of nephrology department. Chin. J. Pharm. 55 (23), 1969–1973. 10.11669/cpj.2020.23.011

[B11] Zhao R. Hang Y. F. Liu X. X. Tan X. Yang Y. Chen L. (2020). Establishment of drug rehabilitation service model for patients with chronic kidney disease in our hospital. Chin. J. Pharm. 55 (5), 408–412. 10.11669/cpj.2020.05.014

