# Supplementary material for: Prevalence and influence factor of drug-related problems in inpatients with kidney disease: a prospective single central study
Source: Front Pharmacol. 2024 Oct 30;15:1436561. doi: 10.3389/fphar.2024.1436561 (PMC11557342; doi:10.3389/fphar.2024.1436561)
Supplement: Supplementary file 1 [file Table1.docx]

**Supplementary Material**

Table S1. The Morisky Medication Adherence Scale (MMAS-8)

| Item | Scores | |
| --- | --- | --- |
| 1.Do you sometimes forget to take your medicine? | Yes(0) | No(1) |
| 2.People sometimes miss taking their medications for reasons other than forgetting. Thinking over the past 2 weeks, were there any days when you did not take your medicine? | Yes(0) | No(1) |
| 3.Have you ever cut back or stopped taking your medication without telling your doctor, because you felt worse when you took it? | Yes(0) | No(1) |
| 4. When you travel or leave home, do you sometimes forget to bring along your medication? | Yes(0) | No(1) |
| 5.Did you take your medicine yesterday? | Yes(1) | No(0) |
| 6.When you feel like your illness is under control, do you sometimes stop taking your medicine? | Yes(0) | No(1) |
| 7.Taking medication every day is a real convenience for some people. Have you ever been hassled about sticking to your treatment plan? | Yes(0) | No(1) |
| 8.How often do you have difficulty remembering to take all your medications? | Never  Once in a while  Sometimes  Usually  All the time | 1  0.75  0.5  0.25  0 |

Table S2. Logistical regression. Patient variables associated with the presence of DRPs.

| Variable | OR(95% CI) | p |
| --- | --- | --- |
| Average age | 1.004(0.992-1.016) | 0.504 |
| Number of people aged ≥ 65 years | 1.284(0.850-1.940) | 0.234 |
| High blood pressure | 1.448(0.923-2.273) | 0.107 |
| Anemia | 1.702(1.146-2.529)* | 0.008 |
| Diabetes | 1.215(0.785-1.879) | 0.382 |
| Hyperuricemia | 1.197(0.710-2.018) | 0.5 |
| Average number of medication types taken | 1.089(1.034-1.147)* | 0.001 |
| Percentage of people with ≥ 5 drugs (%) | 2.415(1.532-3.807)* | <0.001 |

OR: odds ratio; CI: confidence interval; *: P＜0.05

Table S3. Record PE checklist

The RECORD statement for pharmacoepidemiology (RECORD-PE) checklist of items, extended from the STROBE and RECORD statements, which should be reported in non-interventional

pharmacoepidemiological studies using routinely collected health data

| **Item No** | **STROBE items** | **RECORD items** | **RECORD-PE items** | **Page No** |
| --- | --- | --- | --- | --- |
| **Title and abstract** | | | | |
| 1 | (a) Indicate the study’s design with a commonly used term in the title or the abstract.  (b) Provide in the abstract an informative and balanced summary of what was done and what was found. | 1.1: The type of data used should be specified in the title or abstract. When possible, the name of the  databases used should be included. 1.2: If applicable, the geographical region and timeframe within which the study took place should be  reported in the title or abstract.  1.3: If linkage between databases was conducted for the study, this should be clearly stated in the title or abstract. | — | 1-2 |
| **Introduction** | | | | |
| Background rationale | | | | |
| 2 | Explain the scientific background and rationale for the investigation being reported. | — | — | 2-3 |
| Objectives | | | | |
| 3 | State specific objectives, including any prespecified hypotheses. | — | — | 3 |
| **Methods** | | | | |
| Study design | | | | |
| 4 | Present key elements of study design early in the paper. | — | 4.a: Include details of the specific study design (and its features) and report the use of multiple designs if used.  4.b: The use of a diagram(s) is  recommended to illustrate key aspects of the study design(s), including exposure, washout, lag and observation periods,  and covariate definitions as relevant. | 4,11 |
| Setting | | | | |
| 5 | Describe the setting, locations, and  relevant dates, including periods of  recruitment, exposure, follow-up, and data collection. | — | — | 3 |
| Participants | | | | |
| 6 | (a) Cohort study—give the eligibility  criteria, and the sources and methods of  selection of participants. Describe methods of follow-up. Case-control study—give the eligibility criteria, and the sources and  methods of case ascertainment and control selection. Give the rationale for the choice of cases and controls. Cross sectional  study—give the eligibility criteria, and the sources and methods of selection of  participants.  (b) Cohort study—for matched studies,  give matching criteria and number of  exposed and unexposed. Case-control  study—for matched studies, give matching criteria and the number of controls per | 6.1: The methods of study  population selection (such as codes or algorithms used to identify  participants) should be listed in detail. If this is not possible, an explanation should be provided.  6.2: Any validation studies of the codes or algorithms used to select the population should be  referenced. If validation was  conducted for this study and not published elsewhere, detailed methods and results should be provided.  6.3: If the study involved linkage of databases, consider use of a flow | 6.1.a: Describe the study entry criteria and the order in which these criteria  were applied to identify the study  population. Specify whether only users with a specific indication were included and whether patients were allowed to enter the study population once or if  multiple entries were permitted. See explanatory document for guidance related to matched designs. | 3 |

|  | case. | diagram or other graphical display to demonstrate the data linkage  process, including the number of individuals with linked data at each stage. |  |  |
| --- | --- | --- | --- | --- |
| Variables | | | | |
| 7 | Clearly define all outcomes, exposures, predictors, potential confounders, and  effect modifiers. Give diagnostic criteria, if applicable. | 7.1: A completelist of codes and algorithms used to classify  exposures, outcomes, confounders, and effect modifiers should be  provided. If these cannot be  reported, an explanation should be provided. | 7.1.a: Describe how the drug exposure definition was developed.  7.1.b: Specify the data sources from which drug exposure information for individuals was obtained.  7.1.c: Describe the time window(s)  during which an individual is considered exposed to the drug(s). The rationale for selecting a particular time window  should be provided. The extent of  potential left truncation or left censoring should be specified.  7.1.d: Justify how events are attributed to current, prior, ever, or cumulative drug exposure.  7.1.e: When examining drug dose and risk attribution, describe how current, historical or time on therapy are  considered.  7.1.f: Use of any comparator groups  should be outlined and justified.  7.1.g: Outline the approach used to  handle individuals with more than one relevant drug exposure during the study period. | 3-4 |
| Data sources/measurement | | | | |
| 8 | For each variable of interest, give sources of data and details of methods of  assessment (measurement). Describe  comparability of assessment methods if there is more than one group. | — | 8.a: Describe the healthcare system and mechanisms for generating the drug  exposure records. Specify the care  setting in which the drug(s) of interest was prescribed. | 3 |
| Bias | | | | |
| 9 | Describe any efforts to address potential sources of bias. | — | — | 4-5 |
| Study size | | | | |
| 10 | Explain how the study size was arrived at. | — | — | 1 |
| Quantitative variables | | | | |
| 11 | Explain how quantitative variables were handled in the analyses. If applicable, describe which groupings were chosen, and why. | — | — | 3-4 |
| Statistical methods | | | | |
| 12 | (a) Describe all statistical methods, including those used to control for confounding.  (b) Describe any methods used to examine subgroups and interactions.  (c) Explain how missing data were addressed.  (d) Cohort study—if applicable, explain how loss to follow-up was addressed.  Case-control study—if applicable, explain how matching of cases and controls was addressed. Cross sectional study—if | — | 12.1.a: Describe the methods used to evaluate whether the assumptions have been met.  12.1.b: Describe and justify the use of multiple designs, design features, or analytical approaches. | 3-4 |

|  | | applicable, describe analytical methods taking account of sampling strategy.  (e) Describe any sensitivity analyses. | |  | |  | |  |
| --- | --- | --- | --- | --- | --- | --- | --- | --- |
| Data access and cleaning methods | | | | | | | | |
| 12 | | — | | 12.1: Authors should describe the extent to which the investigators had access to the database  population used to create the study population.  12.2: Authors should provide  information on the data cleaning methods used in the study. | | — | | 3-4 |
| Linkage | | | | | | | | |
| 12 | | — | | 12.3: State whether the study  included person level, institutional level, or other data linkage across two or more databases. The  methods of linkage and methods of linkage quality evaluation should be provided. | | — | | 3-4 |
| **Results** | | | | | | | | |
| Participants | | | | | | | | |
| 13 | | (a) Report the numbers of individuals at each stage of the study (eg, numbers  potentially eligible, examined for  eligibility, confirmed eligible, included in the study, completing follow-up, and  analysed).  (b) Give reasons for non-participation at each stage.  (c) Consider use of a flow diagram. | | 13.1: Describe in detail the  selection of the individuals  included in the study (that is, study population selection) including  filtering based on data quality, data availability, and linkage. The  selection of included individuals can be described in the text or by means of the study flow diagram. | | — | | 5-6,11 |
| Descriptive data | | | | | | | | |
| 14 | | (a) Give characteristics of study  participants (eg, demographic, clinical, social) and information on exposures and potential confounders.  (b) Indicate the number of participants with missing data for each variable of interest.  (c) Cohort study—summarise follow-up time (eg, average and total amount). | | — | | — | | 5-6 |
| Outcome data | | | | | | | | |
| 15 | | Cohort study—report numbers of outcome events or summary measures over time. Case-control study—report numbers in  each exposure category, or summary  measures of exposure. Cross sectional  study—report numbers of outcome events or summary measures. | | — | | — | | 5 |
| Main results | | | | | | | | |
| 16 | | (a) Give unadjusted estimates and, if  applicable, confounder adjusted estimates and their precision (eg, 95% confidence intervals). Make clear which confounders were adjusted for and why they were  included.  (b) Report category boundaries when  continuous variables are categorised.  (c) If relevant, consider translating  estimates of relative risk into absolute risk for a meaningful time period. | | — | | — | | 5 |
| Other analyses | | | | | | | |  |
| 17 | Report other analyses done—eg, analyses of subgroups and interactions, and sensitivity analyses. | — | | — | | 5-6 | |  |
| **Discussion** | | | | | | | |  |
| Key results | | | | | | | |  |
| 18 | Summarise key results with reference to study objectives. | — | | — | | 6-8 | |  |
| Limitations | | | | | | | |  |
| 19 | Discuss limitations of the study, taking into account sources of potential bias or imprecision. Discuss both direction and magnitude of any potential bias. | 19.1: Discuss the implications of using data that were not created or collected to answer the specific  research question(s). Include  discussion of misclassification bias, unmeasured confounding, missing data, and changing eligibility over time, as they pertain to the study  being reported. | | 19.1.a: Describe the degree to which the chosen database(s) adequately captures the drug exposure(s) of interest. | | 8-9 | |  |
| Interpretation | | | | | | | |  |
| 20 | Give a cautious overall interpretation of results considering objectives, limitations, multiplicity of analyses, results from  similar studies, and other relevant evidence. | — | | 20.a: Discuss the potential for  confounding by indication,  contraindication or disease severity or selection bias (healthy adherer/sick  stopper) as alternative explanations for the study findings when relevant. **[A: Original text indicated this item was RECORD (ie, not RECORD-PE)?]** | | 8-9 | |  |
| Generalisability | | | | | | | |  |
| 21 | Discuss the generalisability (external validity) of the study results. | — | | — | | 8-9 | |  |
| **Other information** | | | | | | | |  |
| Funding | | | | | | | |  |
| 22 | Give the source of funding and the role of the funders for the present study and, if applicable, for the original study on which the present article is based. | — | | — | | 9 | |  |
| Accessibility of protocol, raw data, and programming code | | | | | | | |  |
| 22 | — | 22.1: Authors should provide  information on how to access any supplemental information such as the study protocol, raw data, or programming code. | | — | | 14-15 | |  |

RECORD=reporting of studies conducted using observational routinely collected data; RECORD-PE=RECORD for

pharmacoepidemiological research; STROBE=strengthening the reporting of observational studies in epidemiology.

*[REFERENCE:Langan SM, Schmidt S, Wing K, Ehrenstein V, Nicholls S, Filion K, Klungel O, Petersen](https://www.bmj.com/content/363/bmj.k3532) [I, Sorensen H, Guttmann A, Harron K, Hemkens L, Moher D, Schneeweiss S, Smeeth L, Sturkenboom](https://www.bmj.com/content/363/bmj.k3532) [M, von Elm E, Wang S, Benchimol EI. The REporting of studies Conducted using Observational](https://www.bmj.com/content/363/bmj.k3532)

[Routinely-collected health Data (RECORD) Statement for Pharmacoepidemiology (RECORD-PE).*BMJ*](https://www.bmj.com/content/363/bmj.k3532) [2018; 363: k3532.](https://www.bmj.com/content/363/bmj.k3532)
